# Bridging minds and policies: supporting early career researchers in translating computational psychiatry research

**DOI:** 10.1038/s41386-024-01834-1

**Published:** 2024-02-28

**Authors:** Aleya A. Marzuki, Tsen Vei Lim

**Affiliations:** 1 Department of Psychology, Sunway University, Petaling Jaya, Selangor Malaysia; 2 Department of Psychiatry and Psychotherapy, Medical School and University Hospital, Eberhard Karls University of Tübingen, Tübingen, Germany; 3 German Center for Mental Health (DZPG), Tübingen, Germany; 4 Department of Psychiatry, University of Cambridge, Cambridge, UK

**Keywords:** Predictive markers, Translational research, Human behaviour, Neuroscience

A significant challenge for psychiatry is to explain precisely how the brain generates psychopathology, as its translation is presumed to advance effective mechanism-based treatments. Computational psychiatry – a mathematical understanding of mental illness – has emerged to bridge this explanatory gap [1]. Broadly, computational psychiatry uses mathematical models to study psychiatric disorders, typically done via 1) an explanatory quantitative modelling approach to explain how aberrant computations of the mind produce psychiatric symptoms, and 2) data-driven modelling, commonly used to predict and track symptom progression. These methods have been applied to identify clinically relevant markers in psychiatry [2–4]. Recently, start-ups have been applying these principles to clinical settings for aiding diagnosis (e.g., https://limbic.ai/) and delivering personalised psychotherapy (e.g., https://alena.com/).

Early career researchers (ECRs) are uniquely positioned to advance the translation of computational psychiatry. However, during our own academic training, we encountered barriers that may limit its uptake amongst ECRs. Here, we highlight these barriers and propose potential solutions.

## Accessibility

Most mental health researchers are not mathematically trained. Computational psychiatry requires foundational knowledge in statistical modelling and programming for its application or understanding. However, such training is not routinely embedded into postgraduate programs. Most computational psychiatry studies are conducted by institutions with strong computational foundations and training capacity, often within Western, Educated, Industrial, Rich, Democratic (WEIRD) settings.

To address accessibility, we propose three potential solutions. One, mental health research labs should consider inter-disciplinary collaborations with researchers who have necessary quantitative skills (e.g., data scientists, computer scientists) to manage complex data. This would not only promote mutual learning and crosstalk between psychiatry, psychology, and computational neuroscience, but also enhance research efficiency (i.e., obviating the need to learn complex analyses from scratch). Two, upskilling ECRs in quantitative skills should be prioritised. Universities could embed programming and quantitative courses into psychology-based programs to cater for those interested in pursuing computational psychiatry (e.g., undergraduate subjects on computational modelling, MSc in computational cognitive science). Code-sharing alongside publications should be encouraged as they are invaluable learning resources for ECRs. Three, upskilling ECRs should involve researchers from the Global South, as the development of translatable computational models should not ignore cross-cultural validity. High-income countries could collaborate, or initiate specific schemes to encourage sharing of knowledge and resources with the Global South (e.g. the Oxford University Wellcome Centre Integrative Neuroimaging Global Scholars (WINGS) programme for researchers from countries with limited research investment).

## Translatability

Although translational computational psychiatry seems promising, there are still significant ways to go. One notable limitation is the poor psychometric properties of computational parameters, such as limited construct validity (the extent to which a test measures the intended construct) and test-retest reliability (rate of agreement between measurements taken from the same individual at different timepoints) [5]. These problems significantly impede the reliability of computational parameters as markers for psychiatry, which in part explains why these parameters are not incorporated in routine clinical practice [6]. Furthermore, although psychiatry research recognizes sociocultural factors as determinants of mental ill health, present work in the field rarely incorporates these factors into computational models of mental health [7]. Indeed, accounting for sociocultural considerations has proven successful in adjacent fields like clinical psychology (e.g., incorporating ethnicity and neighbourhood disadvantage into models better explains psychological well-being [8] and psychiatric service usage [9]).

To enhance translatability, we must improve the psychometric properties of computational psychiatry models. Computational findings must always be validated with simulations and parameter recovery [10]. Next, clinically-trained ECRs could also administer behavioural paradigms alongside clinical care to verify the relevance of computational constructs to symptomatology and treatment responses, thereby accelerating their uptake in clinical settings. Similarly, ECRs should be encouraged to collaborate with healthcare sectors, start-ups, and think-tanks to enhance the policy relevance of their research. Academic-industry partnerships could be incorporated into PhD training programs, e.g., the Medical Research Council’s (MRC) industrial Collaborative Awards in Science and Engineering (iCASE) studentship that includes brief collaborations with non-academic partners.

Simultaneously, incorporating patient-public involvement (PPI) in computational psychiatry research can ensure that outcomes have clear clinical relevance from conception. PPI’s benefits are three-fold: 1) it can better inform models or tasks to enhance specificity; 2) it serves as a platform to check model assumptions in ethnically and socioeconomically diverse patient groups; 3) it allows for examination of whether tasks are meaningful and comprehensible to patients. As an example for point 3, the well-known two-step reinforcement learning task, conceived as computational proxies for goal-directed actions and habits in psychiatry [11], might be over-interpreted as it is highly instruction-dependent [12]. Indeed, almost all participants became more goal-directed/model-based when instructions were made more explicit [12] – something that may have been detected earlier with PPI. To facilitate PPI efforts, we advocate for training ECRs in science communication and outreach early on [13, 14], equipping them with skills necessary to communicate research effectively to non-specialist PPI panels.

## Funding

Psychiatry is complex and dynamic. For computational psychiatry to be informative (e.g., identifying markers), there is a need for longitudinal studies with large samples and multi-disciplinary collaborations. Multi-site consortia-based research (e.g., IMAGEN, ENIGMA, and ABCD) have proven that large collaborations over longer timescales can be successful, albeit requires substantial funding. Existing major funders of psychiatry research often offer fellowships and grants for shorter timescales (i.e., 2–5 years), and are highly competitive, which may inadvertently promote “quantity over quality” practices to maintain competitiveness.

High quality and translatable computational psychiatry work requires slow rigorous research, as developing robust paradigms and meaningful models of mental health incurs significant time and resources. Funders should promote ‘slow science’, emphasising quality over quantity and allow for greater timescales for research that advances the field [15], e.g., more longitudinal research. To support ECRs, funding bodies could consider 1) moving away from publication-based milestones and instead emphasise clinical and societal impact, and 2) providing structured mentorship opportunities with established computational psychiatrists.

In conclusion, ECRs form the future for computational psychiatry, but ensuring high-quality work requires support from labs, research institutions, and funders. To play our part, we have curated a list of resources for ECRs interested in delving into computational psychiatry.
